# Black shank-mediated alteration of the community assembly of rhizosphere soil bacteria in tobacco

**DOI:** 10.3389/fmicb.2024.1428284

**Published:** 2024-10-23

**Authors:** Junchi Ma, Jili Chen, Qing Zhang, Yumei Dong, Zhihua Li, Junqiu Xie, Dongmei Yang, Lequn Zhou, Dahao Yan, Bo Zhou, Tao Liu

**Affiliations:** ^1^College of Agronomy and Biotechnology, Yunnan Agricultural University, Kunming, China; ^2^Tobacco Leaf Quality Inspection Section, Raw Material Department, Hongyun Honghe Tobacco (Group) Co., Ltd., Kunming, China; ^3^Pharmaron Beijing Co., Ltd., Beijing, China; ^4^Technology Center of China Tobacco Yunnan Industrial Co., Ltd., Kunming, China

**Keywords:** tobacco, rhizosphere soil bacteria, disease resistance, soil-borne legacy, bacterial diversity

## Abstract

**Introduction:**

There is a close and complex interaction between the elements in the aboveground-underground ecosystem during the growth and development of plants. Specifically, when the aboveground part of plants is infected by pathogens, it induces the plant rhizosphere to synthesize specific root exudates. Consequently, a group of beneficial rhizosphere soil bacteria is recruited to help plants resist diseases. However, the changes in the rhizosphere soil bacterial community of plants under infection by oomycete pathogens remain unknown.

**Methods:**

Three experimental treatments were set up in this experiment: soils inoculated with *P. nicotianae*, no-inoculation with *P. nicotianae*, and a control. The control treatment was composed of soils without transplanted tobacco plants, with the pathogen inoculated twice at an interval of eight days to ensure a successful *P. nicotianae* infection. *P. nicotianae* inoculation treatments were designed using the hyphal block inoculation method. In the non-inoculation treatment, tobacco plants were grown normally without pathogen inoculation. The tobacco plants were grown in a greenhouse.

**Results:**

This study demonstrates that tobacco plants recruit microorganisms at the rhizosphere level as a defense mechanism against disease after infection by the oomycete pathogen *Phytophthora nicotianae*. Specific rhizosphere soil bacteria were screened *in vitro* to promote tobacco growth in a biofilm-forming manner, which induced the systemic resistance of the plants to *P. nicotianae*. The recruitment of rhizosphere soil bacteria to the inter-root zone of tobacco plants after infection by *P. nicotianae* can help subsequently cultivated tobacco plants in the same soil resist pathogen infestation.

**Discussion:**

In conclusion, the present study confirms that infestation caused by oomycete pathogens alters the composition of the plant rhizosphere soil bacterial community and recruits a specific group of beneficial microorganisms that induce disease resistance and promote plant growth, thereby maximizing the protection of progeny grown in the same soil against the disease.

## 1 Introduction

There are close and complex interactions among the elements in the aboveground-underground ecosystem of plants, with different elements interacting through the exchange of materials and information (Raven, 2022; Rodriguez et al., 2019). Studies have shown that the ability of plant root soil to inhibit pathogens is enhanced after a disease outbreak (Weller et al., 2002; Berendsen et al., 2012). When plants are attacked by pathogens, they secrete specific chemicals in their roots, allowing the recruitment of particular bacterial communities that help host plants resist diseases (Lakshmanan et al., 2013; Haney et al., 2015; Pérez-Jaramillo et al., 2017; Pieterse et al., 2016; Wintermans et al., 2016). Pathogen infection (Chapelle et al., 2016; Fernández-González et al., 2020) is the most important external pressure affecting changes in the plant rhizosphere bacterial community. Plants change their gene expression and root exudates after pathogen infection. The beneficial bacteria recruited in the roots of infected plants act as a “call for help” strategy to help plants resist diseases (Stringlis et al., 2018). Some plants can recruit beneficial bacteria by releasing volatile organic compounds (VOCs) (Liu et al., 2019; Schulz-Bohm et al., 2018; Bakker et al., 2018).

During plant growth, plant rhizosphere soil accumulates pathogens, resulting in negative effects on plant growth and development, known as soil negative feedback (Van der Putten et al., 1993; Bever et al., 2012). Although the accumulation of soil-borne pathogens causes severe problems in plant growth and development under monoculture, crop rotation can alleviate this process. For example, planting beets and wheat in rotation may enhance the inhibition of soil on pathogens and improve the disease resistance of plants (Mendes et al., 2011; Raaijmakers and Mazzola, 2016; Weller et al., 2002). Despite pathogens in such disease-suppressive soils, specific soil bacterial communities that inhibit the growth and activities of pathogens keep plants healthy. This phenomenon is usually attributed to the production of antimicrobial compounds that selectively inhibit the growth of pathogens (Mendes et al., 2011; Weller et al., 2002; Raaijmakers and Weller, 1998). Stimulating the host plant immune system by protective rhizosphere soil microorganisms (induced system resistance—ISR) is a potential activation defense mechanism that helps plants resist diseases (Pieterse et al., 2014).

The pathogen of black shank tobacco, *Phytophthora nicotianae* is a representative of oomycetes. *P.nicotianae* is a parasitic *Phytophthora* with a wide host range and is a worldwide disease. The changes in the community of plant rhizosphere soil bacteria under oomycete pathogen infection have not been determined by previous studies. This study selected susceptible cultivar tobacco plants of the Hongda variety as the research material, and these plants were infected by *P. nicotianae*. After one or 2 weeks of treatment, the inter-root and non-inter-root soils of healthy and pathogen-infected plants were collected. This was done to explore the changes in the rhizosphere bacterial community of tobacco under pathogen infection. Furthermore, specific inter-root soil bacteria were screened to investigate the biological relevance of their growth-promoting and disease-resistant effects on host plants. This study aimed to lay a research foundation for subsequent crop cultivation improvement by studying the soil bacterial community in tobacco inter-roots. This kind of research is paramount in exploring a new and stable biocontrol method against *P. nicotianae* and in revealing the mechanism of the biocontrol strains.

## 2 Materials and methods

### 2.1 Plant and soil materials

The Yunnan Tobacco Monopoly Bureau provided tobacco seeds. Tobacco seeds were cultivated with a seedling tray to the seedling stage and then transplanted for planting. The soil was purchased from the Doonan Flower Market, Kunming, Yunnan Province, and sterilized at 121°C in an autoclave for 2 h before use. Sterilized soil was watered to saturation in pots before the experiment. Next, the soil was inoculated with tobacco seedlings. The plants were removed after 3 weeks of growth. Then, the soil was sieved and stored in a refrigerator at 4°C. Finally, the experiment began within 2 weeks.

### 2.2 Experimental treatment

Three experimental treatments were set up in this experiment: soils inoculated with *P. nicotianae*, no-inoculation with *P. nicotianae*, and a control. The control treatment was composed of soils without transplanted tobacco plants, with the pathogen inoculated twice at an interval of 8 days to ensure a successful *P. nicotianae* infection. *P. nicotianae* inoculation treatments were designed using the hyphal block inoculation method (Lin, 1982). The tobacco stems were scratched with a blade, and *P. nicotianae* disks were placed on the cut area of the tobacco. The disks were moisturized with sterile water and covered with skimmed cotto. In the non-inoculation treatment, tobacco plants were grown normally without pathogen inoculation. The tobacco plants were grown in a greenhouse at Yunnan Agricultural University.

### 2.3 Bacteria community analysis

This study focused on using 16S rRNA amplicon sequencing to determine the species and abundance of bacteria. Three pot samples were taken from each treatment on days 8 and 15 after the above experimental treatments to analyze the bacterial community. This procedure was performed to collect the soil body (non-inter-root soil portion in potted plants) and the inter-root soil (50–250 mg) for bacterial community analysis. The soil was collected and stored at low temperatures and sent to Wuhan Kangtest Technology Co. for rhizosphere soil bacteria diversity testing on the same day.

### 2.4 Isolation of inter-root soil bacteria

The tobacco rhizosphere soil infected by tobacco pathogens was stored in a − 80°C refrigerator at the end of the above experiment. The rhizosphere soil was thawed at room temperature. Next, 10 g of soil was weighed and placed in a culture bottle containing 90 mL of sterile water. The soil suspension was prepared by sealing with a sealing film and oscillating at 37°C, 180 rpm for 2 h. Then, a soil suspension was prepared. The soil suspension was allowed to stand for 5 min and transferred to a centrifuge tube. Next, 1 mL of supernatant was obtained after centrifugation and diluted it. The dilutions were inoculated on the following media to maximize the isolation of culturable bacterial species: (1) tryptone soy agar (TSA) medium, (2) nutrient agar medium, (3) peptone yeas agar medium, (4) R_2_A medium, (5) King’s B medium, (6) Luria-Bertani (LB) medium. After inoculation, they were incubated at 30°C for 1–2 d. Each gradient was repeated three times while a control was set. Single colonies with different morphologies and colors were selected. Single colonies were inoculated on an LB solid culture plate for purification, and the purified bacteria were cultured in a bacterial liquid with an LB liquid medium. Next, 1 mL of bacterial liquid was aspirated, mixed with 50% sterilized glycerol at a ratio of 1:1, and preserved at −80°C for spare use.

### 2.5 Amplification and sequencing of bacterial 16SrRNA

The bacterial 16S rRNA gene was amplified and sequenced for molecular identification. Endophyte genomic DNA was extracted using the CTAB method (Kosecka-Strojek et al., 2019). The primer sequences used for PCR identification were 27F (5’-GAGTTTGATCACTGGCTCAG-3′) and 1492R (5’-TACGGCTACCTTGTTACGA-3′) (Hassan, 2017). The PCR reaction system comprised 22.5 μL of gold MIX, 1 μL each of F and R, and 0.5 μL of genome template. The PCR amplification system was as follows: pre-denaturation at 98°C for 2 min, denaturation at 98°C for 10 s, annealing at 55°C for 30 s, extension at 72°C for 10 s, 30 cycles, and extension at 72°C for 5 min was used after this procedure.

After the reaction, the products were obtained and evaluated for amplification by agarose gel electrophoresis. The quality of the products was assessed according to the results and sent to Kunming Shuoqin Biotechnology Co. for sequencing. The sequencing results were compared with blast in the NCBI database. The homologous sequences were searched to find the strain with the highest similarity and determine its biological classification status.

### 2.6 Quantification of biofilm formation

Biofilms isolated from selected bacteria were quantified following the method of Ren et al. (2015). The three isolated bacterial strains were inoculated in 5 mL of LB liquid medium and incubated at 20°C, 180 rpm for 24 h. Subsequently, they were diluted with LB liquid medium, transferred to 5 mL of LB liquid medium, and re-incubated at 20°C, 180 rpm overnight.

The absorbance of the bacterial solution (OD590) was measured at 590 nm as an exponential increase of approximately 0.5. It was diluted to an OD of 1.5 using an LB liquid medium. The bacterial cultures were then added to the 96-well plates alone or in equal mixes with other cultures. The control was set up as an LB liquid medium with a total volume of 160 μL. Cells were sealed using a 96-well silica gel lid and incubated at 20°C for 48 h at rest, and unfixed cells were rinsed with 200 μL of phosphate-buffered salt solution (PBS) after incubation. The cells were washed, and the biofilms were stained with 180 μL of crystal violet containing 1% water (W/V) for 20 min. After the staining was completed, the cells were rinsed three more times with PBS buffer and incubated with 200 μL of 96% ethanol to release the crystal violet absorbed by the biofilm. Finally, the OD value of the crystalline violet ethanol solution (OD 590 nm) was determined using an enzyme marker to quantify the thickness of the biofilm.

### 2.7 Synergy between colonies

The isolated bacteria were inoculated in 5 mL of Kim’s B liquid medium and incubated overnight at 20°C, 180rm. The bacterial culture was adjusted at an optical density of 600 nm (OD600 nm) to 0.1 using a multi-channel pipette to 1 μL. The diluted bacterial solution was inoculated on the diagonals of both sides of a square Petri dish poured with Kim’s medium, inoculated with 10 points, showing a “V” shape with the growth of bacteria, and then incubated in a 20°C incubator for 15 days after covering and sealing. Observe the distance between the colony morphology and community diameter and the vertical direction of the “V” line.

### 2.8 Selection of rifampicin-resistant mutants

Rifampicin-resistant strains were screened according to the method of Glandorf et al. (1992) to quantify the number of bacteria in plant rhizosphere soil during plant growth and ISR determination. In brief, the colonies of these strains were transferred to TSA agar plates containing increasing concentrations (50, 100, 150, 200, and 250 μg/mL) of rifampicin.

### 2.9 Growth-promoting experiment

#### 2.9.1 Inoculation of rhizosphere soil bacteria

Preparation of bacterial suspension: the screened bacteria were inoculated into 250 mL of LB liquid medium and incubated at 20°C, 180 rpm for 1–2 days. The OD600 value of the bacterial suspension was determined, and the bacterial suspension was obtained when the OD600 equaled 0.8 (about 1 × 10^8^ cfu/g). The bacterial suspension was collected by centrifuging the bacterial suspension at 5000 rpm, 4°C for 20 min, and then stored at 4°C for future use.

A rhizosphere bacterium, *Bacillus cereus*, numbered lgd2 (Yan et al., 2022), was identified as having a growth-promoting and disease-resistant effect on tobacco, was selected. The rifampicin-resistant mutants, *Arthrobacter* sp., *Pseudomonas* sp., and *Massilia* sp., were individually or mixed to make a soil suspension with a density of 1 × 10^8^ cfu/g bacterial suspension was inoculated in the tobacco inter-root soil.

#### 2.9.2 Analysis of the parameters of the net photosynthetic rate of tobacco agronomic traits

Agronomic traits, such as tobacco plant height, stem width, leaf length, and leaf width, were measured with a straightedge (accuracy of 0.1 cm) and vernier calipers (accuracy of 0.1 mm) at harvest after 6 weeks of plant growth. Use an electronic balance (accurate to 0.1 g) to determine the fresh weight of the aboveground part of the tobacco plants. A Li-6400 XT photosynthetic fluorescence recorder and a red and blue light source leaf chamber were used. The light quantum flux density was 300 umol/ (M2•s), the temperature was 25°C, and the CO_2_ concentration was 400 umol/mol. Three plants were selected for each treatment, and one leaf was randomly selected for each plant to determine the net photosynthetic rate (Pn).

### 2.10 ISR analysis

In the ISR determination, the plant growth mode and experimental treatment were similar to the growth promotion experiment. The incidence of each treatment was counted. The disease degree was quantified by measuring the number of spores of tobacco black shank disease according to the method of Van Damme et al. (2005): after 6 weeks of tobacco growth, the infected part of tobacco was collected and placed in a centrifuge tube containing 10 mL distilled water, vortexed for 30 s, and then the spores were counted using a microscope. The number of *P. nicotianae* spores produced by each plant was then calculated and repeated three times.

### 2.11 Formation of a soil-borne legacy

This study refers to the method of Berendsen et al. (2018) to determine the soil heritage. This procedure was performed to determine whether tobacco recruits specific bacteria in the rhizosphere to help it resist diseases after being infected by pathogens and whether it can help the next generation of plants resist diseases after forming a soil heritage. For soil treatment, as mentioned above, in each of the 40 pots filled with 3 kg of soil watered to saturation, tobacco seeds were soaked in darkness at 4°C for 2 days. One seed per pot was sown in 20 pots, and the remaining pots were left unseeded. The plants were watered with half the Hoagland’s nutrient solution concentration and then transferred to the greenhouse for incubation.

Ten pots of plants were selected and inoculated with *P. nicotianae* using the mycelial block inoculation method, covered with skimmed cotton for moisturization when the tobacco seeds germinated and grew to four-leaf age. The remaining 10 pots were sprayed with sterile water. Ten pots not planted with tobacco seeds were selected and sprayed with tobacco blight spore suspension. The remaining 10 pots were sprayed with sterile water treatment. Two weeks after the infection of the tobacco black shank, the aboveground parts of the first batch of plants were cut off to remove the underground parts, and new tobacco seeds were sown in all flowerpots. The second batch of tobacco plants was inoculated with pathogens or treated with water for simulation after 2 weeks of growth. After 2 weeks of pathogen infection, the number of pathogen spores produced in each pot of aboveground plant tissue was determined using the above method to quantify the severity of black shank disease.

### 2.12 Data processing

The obtained test data were statistically analyzed using Excel 2019 software. IBM SPSS Statistics 26 (SPSS, Chicago, United States) analysis software was used for the statistical analysis. A one-way ANOVA was used for the significance analysis. Duncan’s multiple comparison method was used to analyze the difference in significance between treatments. Origin2022 software was used for mapping.

## 3 Results

### 3.1 Inter-root soil bacteria community changes on immune-stimulated plants

**Figure 1:**
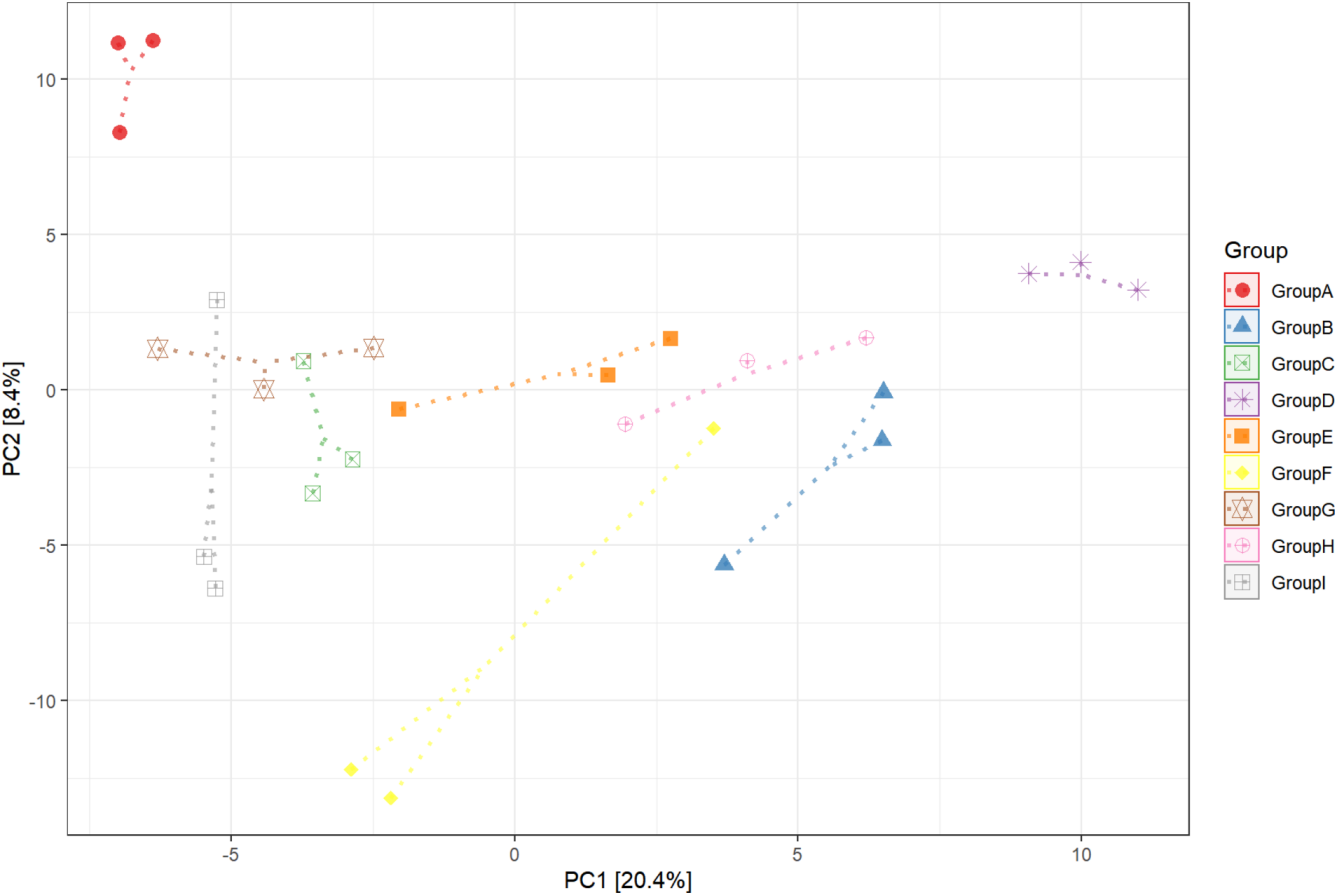
Principal component (PC) analysis of rhizosphere soil bacterial communities in unplanted soil, infected by pathogens, and healthy tobacco. CK refers to non-planted soil; N15d is the non-rhizosphere soil of tobacco on the 15th day of health; N15dg is the tobacco rhizosphere soil on the 15th day of health; N8d is the non-rhizosphere soil of tobacco on the eighth day of health; N8dg is the tobacco rhizosphere soil on the eighth day of health; YM15d is the non-rhizosphere soil of tobacco on the 15th day after pathogen infection; YM15dg is the tobacco rhizosphere soil on the 15th day after being infected with the pathogen; YM8d is the non-rhizosphere soil of tobacco on the eighth day after being infected with the pathogen; YM8dg is the tobacco rhizosphere soil on the eighth day after being infected with the pathogen.

This experiment observed the changes in the bacterial community in the rhizosphere tobacco soil within 15 days to study the effect of *P. nicotianae* infection on the bacterial community induced by the roots soil of tobacco plants. A principal component analysis was used in this experiment to identify the key drivers of bacterial abundance variability in samples (Figure 1). There were significant differences in the composition of bacterial communities in the tobacco rhizosphere soil after infection with pathogens. The first principal component (PC1 explained 20.4% of the variance variation) separated the rhizosphere of plants infected by the pathogen tobacco black shank from other rhizospheres. The second principal component (PC2 explained 8.4% of the variance variation) separated soil samples without tobacco cultivation from soil samples with tobacco cultivation. The bacterial community between pathogen-infected tobacco and healthy tobacco non-rhizosphere soil did not appear to have obvious separation. Still, the soil bacteria community without tobacco was separated, indicating that planting tobacco would change the soil bacteria community. After eight and 15 days of *P. nicotianae* infection of tobacco, the bacterial community in the rhizosphere soil of tobacco was considerably separated from that of healthy tobacco. The rhizosphere soil of non-cultivated plants and the bacterial community distribution in the rhizosphere soil of tobacco infected with *P. nicotianae* were also considerably different from that of tobacco infected with *P. nicotianae* for eight and 15 days (Supplementary Figure S1). This result indicated that the increase in infection time affected the changes in bacterial diversity in the rhizosphere soil of tobacco under the infection of *P. nicotianae*.

### 3.2 Characteristics of the rhizosphere bacterial community recruited by pathogen-infected plants

**Figure 2:**
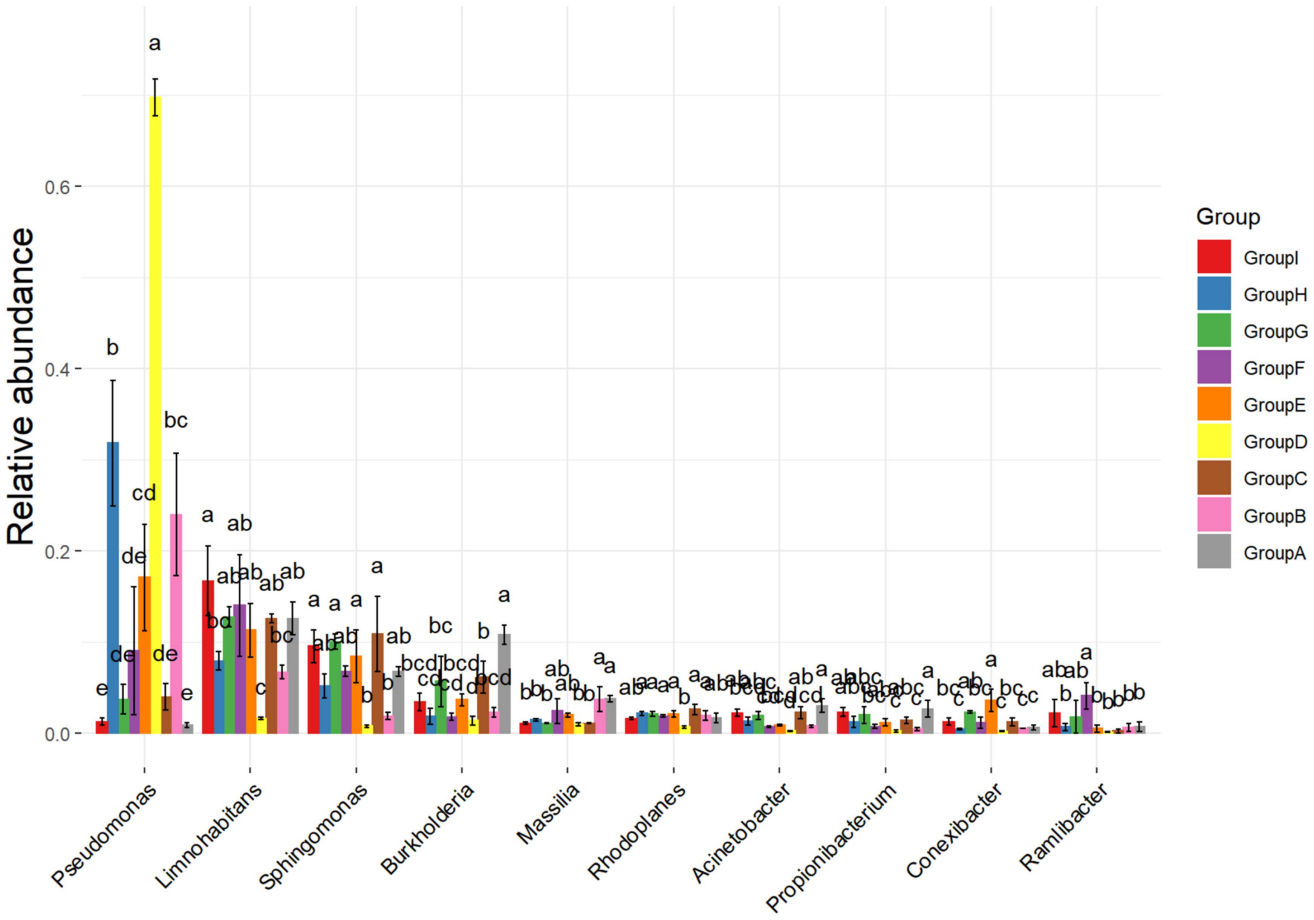
Analysis of differences in bacterial communities. The ANOVA parameter method was used to analyze species richness data between groups, which is suitable for difference analysis under the conditions of normal distribution and regular variance.

In this experiment, two rhizosphere soil bacteria were selected from the 10 high-ranking abundance bacteria in tobacco rhizosphere soil (Figure 2). One *Pseudomonas* sp. strain (hereinafter referred to as P.) was significantly higher in the rhizosphere enrichment abundance after *P. nicotianae* infection than the uninfected bacterium. Another strain was *Massilia* sp. (hereinafter referred to as M.), a high-abundance bacterium with an antagonistic effect on *P. nicotianae*. The last low-abundance bacterium was *Arthrobacter* sp. (hereinafter referred to as A.) (Supplementary Figure S2). Still, its abundance was significantly higher than that of uninfected tobacco after infection with *P. nicotianae*. Two hundred twenty-four rhizosphere soil bacteria, representing 35 genera, were isolated and identified from the rhizosphere soil of tobacco plants infected with pathogens using different isolation methods to further determine the function of rhizosphere soil bacteria. *Pseudomonas* sp. (accession number: OR053801), *Massilia* sp. (accession number: OR053802), and *Arthrobacter* sp. (accession number: OR053800) were isolated to form a bacterial consortium for subsequent research (Supplementary Table S1). A confrontation test showed that the above three bacterial strains resisted *P. nicotianae* antagonism.

### 3.3 Prediction of rhizosphere soil bacteria function

**Figure 3:**
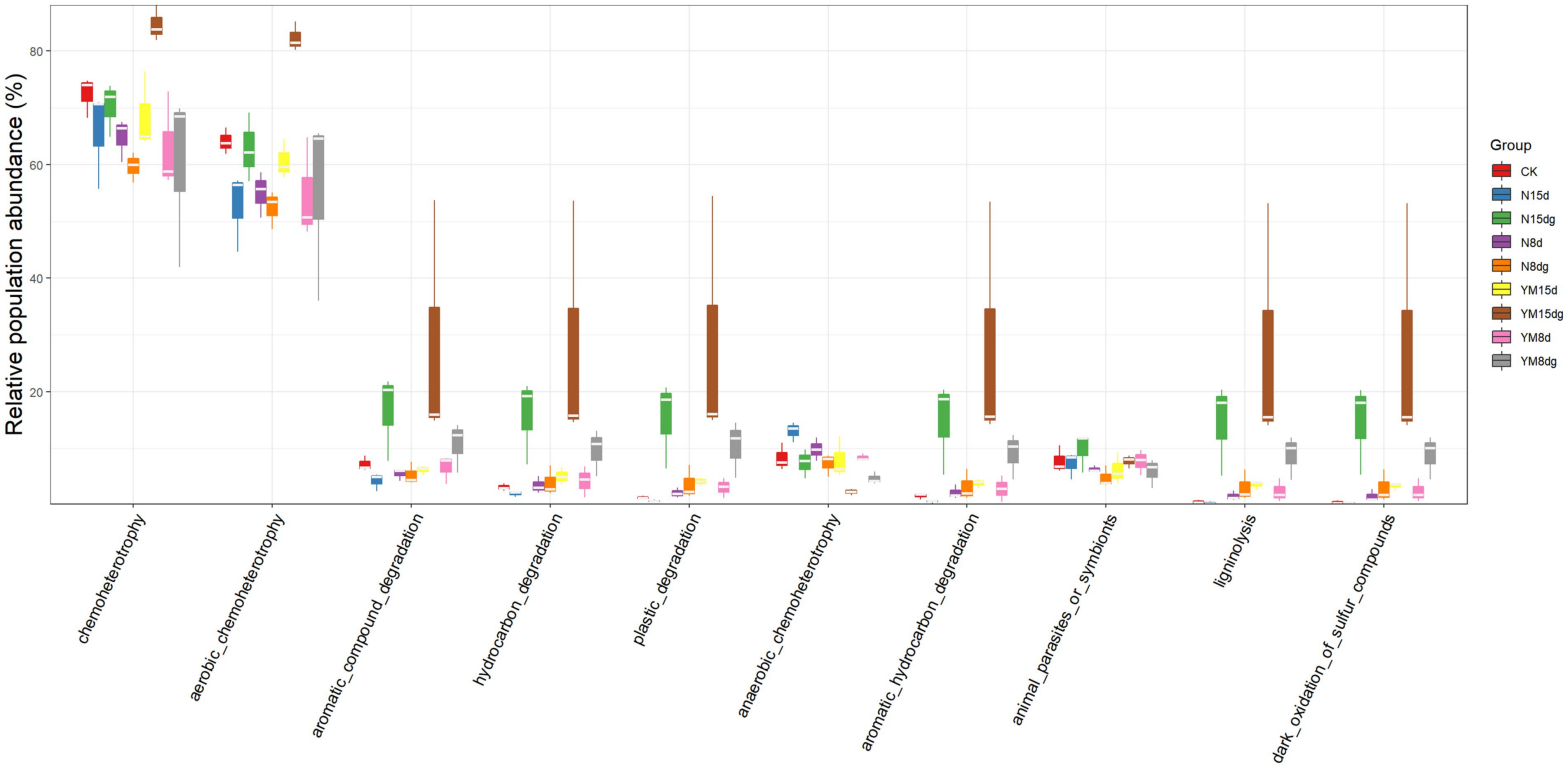
Prediction of rhizosphere soil bacterial function. FAPROTAX software maintains a functional classification database based on species information. The function of faprotax prediction focuses on the function of microorganisms in oceans and lakes. These functions include more than 80 functional classifications, such as carbon, nitrogen, phosphorus, sulfur, and other elements circulation, animal and plant pathogens, methane generation, and fermentation. FAPROTAX covers more than 4,600 different prokaryotic species and has a good prediction effect on the biochemical cycle process of environmental samples.

It is a good choice to use amplicon sequencing data to predict the functional spectrum when determining the function of each out. In this experiment, FAPROTAX software was used to indicate the functions of tobacco rhizosphere soil bacteria (Figure 3). After the pathogen infects tobacco, the main function of bacteria enriched in the tobacco rhizosphere soil is to provide chemical fertilizer for plant growth. This result indicates that plant rhizosphere soil bacteria promote plant growth (Kloepper and Schroth, 1978). After being infected by pathogens, the abundance of rhizosphere soil bacteria that provide chemical fertilizers for plant growth increased over time compared with plants not infected by pathogens. The functions of other rhizosphere soil bacteria are the degradation of aromatic compounds, hydrocarbons, and microplastics. These bacteria can directly or indirectly help plant growth and development as biological fertilizers.

The abundance of bacteria that function to degrade aromatic compounds, hydrocarbons, and microplastics increases significantly in the plant rhizosphere soil with an increase in plant growth time. Still, the content of bacteria that promote plant growth and development under pathogen infection is significantly higher than that of healthy plants. In conclusion, the number of rhizosphere soil bacteria contributing to plant growth increased significantly with a long plant growth time, promoting plant growth. In addition, the number of growth-promoting bacteria was high in the case of pathogen infection.

### 3.4 Beneficial effects of recruited rhizosphere soil bacteria on plants

**Figure 4:**
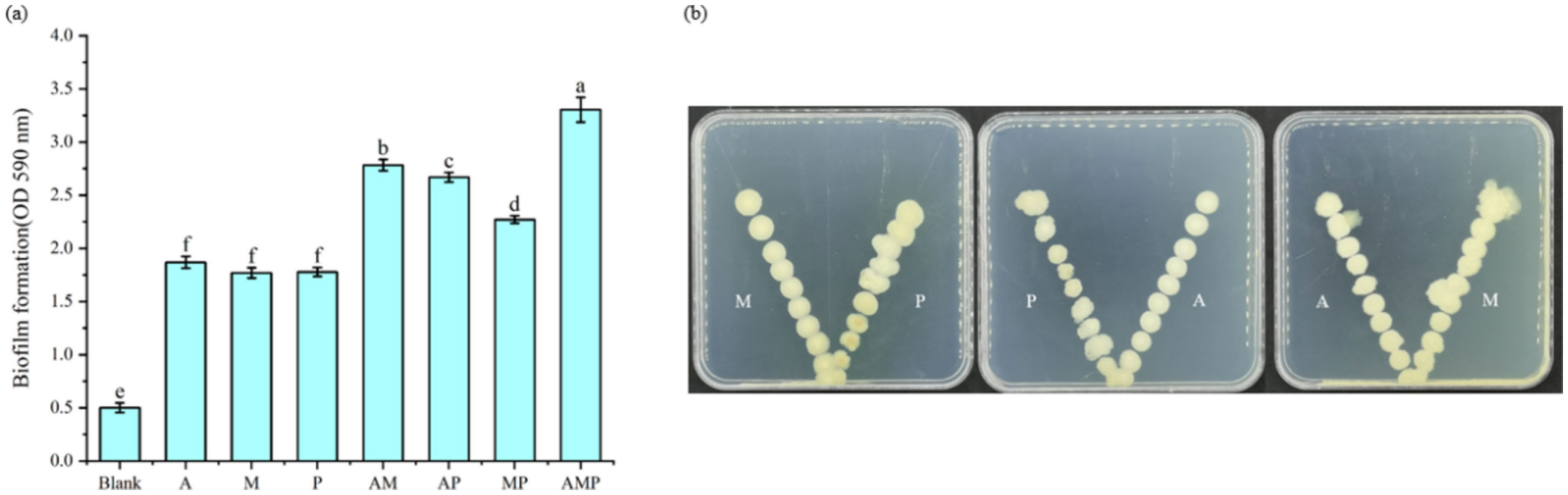
Interactions between rhizosphere soil bacteria. M: *Massilia* sp., P: *Pseudomonas* sp., and A: *Arthrobacter* sp. The rest are a mixture of two or three strains of bacteria. **(A)** Indicates the thickness of Biofilm formation at OD590 nm, **(B)** Indicates the synergism between two of the three bacterial strains.

The recruitment of three selected rhizosphere soil bacterial strains as a consortium suggests they are associated with plant roots. These rhizosphere-colonizing bacteria form biofilms on the root surface, triggering a variety of microbial processes, such as the production of plant growth regulators, defense inducers, and antibiotics, and affecting the host and other rhizosphere microorganisms (Beauregard et al., 2013). The three isolated rhizosphere soil bacteria were cultured *in vitro* to study whether the three strains of rhizosphere soil bacteria played a synergistic role in biofilm formation. The experiment proved that the three strains of rhizosphere soil bacteria played a synergistic role in biofilm formation. Specifically, the biofilm formed by combining these three strains of rhizosphere soil bacteria was always greater than that formed by individual strains (Figure 4A). This result indicates that when pathogens infect tobacco, three rhizosphere soil bacteria are recruited in the tobacco rhizosphere as a consortium. At the same time, these three strains of bacteria attracted each other to the plate (Figure 4B). These three rhizosphere soil bacteria, *Pseudomonas* sp., *Massilia* sp., and *Arthrobacter* sp., did not show an antagonistic effect on the plate and would promote the growth of another bacterium near a separate colony.

**Figure 5:**
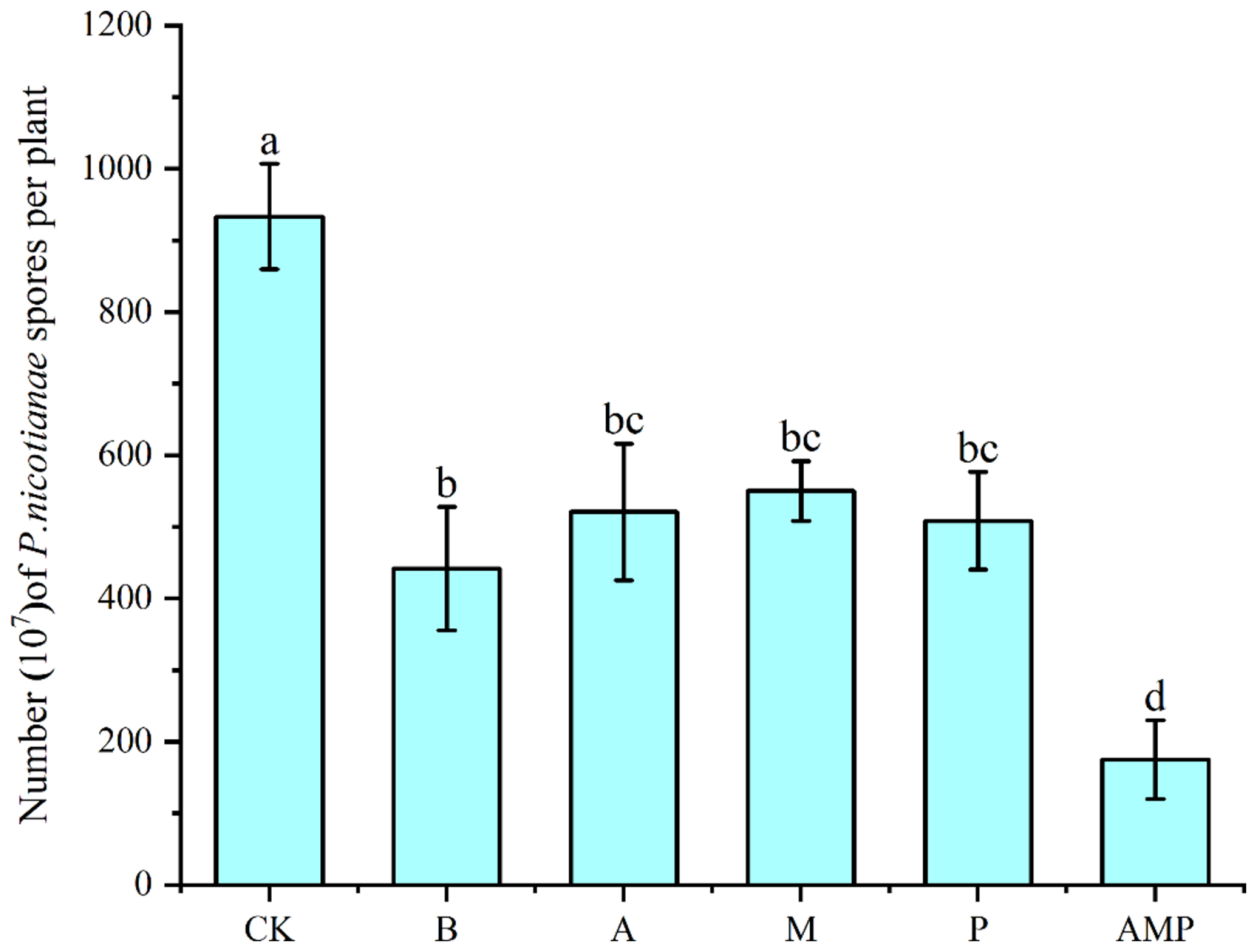
Number of pathogen spores. CK was not inoculated with inter-root soil bacteria; B: *Bacillus cereus*, A: *Arthrobacter* sp., M: *Massilia* sp., P: *Pseudomonas* sp., APM is a consortium of three bacterial strains.

We rewired the three bacteria alone or their consortium into the tobacco rhizosphere soil infected by *P. nicotianae* to evaluate their ability to induce the ISR pathway against pathogen infection. This procedure was performed to study the biological relevance of the consortium of the three rhizosphere soil bacteria. *Bacillus cereus* number lgd2 (hereinafter B.) is a rhizosphere soil bacterium confirmed to help tobacco resist black shank disease and promote growth (Yan et al., 2022). The colonization of tobacco rhizosphere soil by strain B induced the ISR pathway, just as the number of spores in tobacco disease spots significantly decreased after pathogen infection (Figure 5). Colonizing a single strain can significantly reduce the number of pathogen spores on infected plants, and the combination of three strains is better than a single strain. The colonization of a combination of three rhizosphere soil bacteria in the rhizosphere significantly reduced the number of spores of pathogens compared with the colonization of a single strain. In conclusion, tobacco plants recruit three rhizosphere soil bacteria after pathogens infect plants: *Pseudomonas* sp., *Massilia* sp., and *Arthrobacter* sp. These strains can systematically enhance plant protection. These results support the view that, as a consortium, rhizosphere soil bacteria help host plants resist diseases.

**Figure 6:**
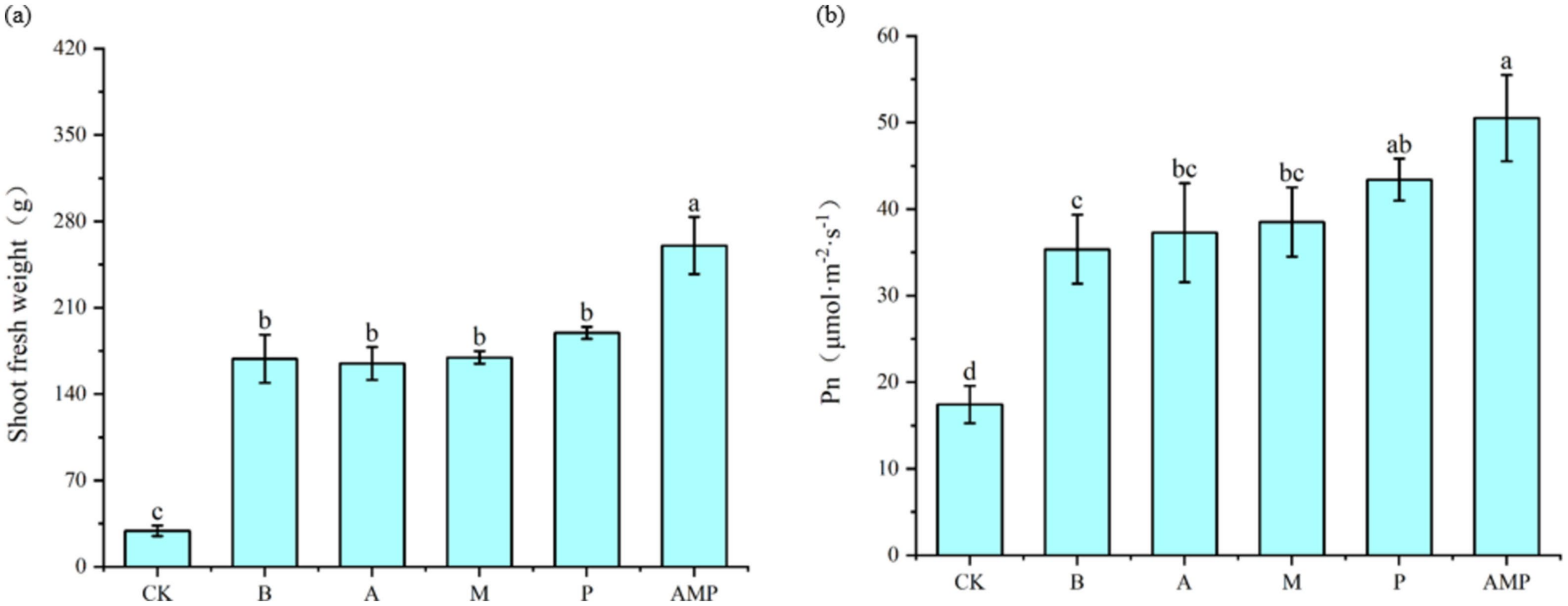
Effects of rhizosphere soil bacteria on the net photosynthetic rate and biomass of tobacco. CK was not inoculated with inter-root soil bacteria; B: *Bacillus cereus*, A: *Arthrobacter* sp., M: *Massilia* sp., P: *Pseudomonas* sp., APM is a consortium of three bacterial strains. **(A)** Indicates the effect of the combination of three inter-root soil bacteria on tobacco biomass. **(B)** Represents the effect of the combination of three inter-root soil bacteria on the net photosynthetic rate of tobacco.

**Figure 7:**
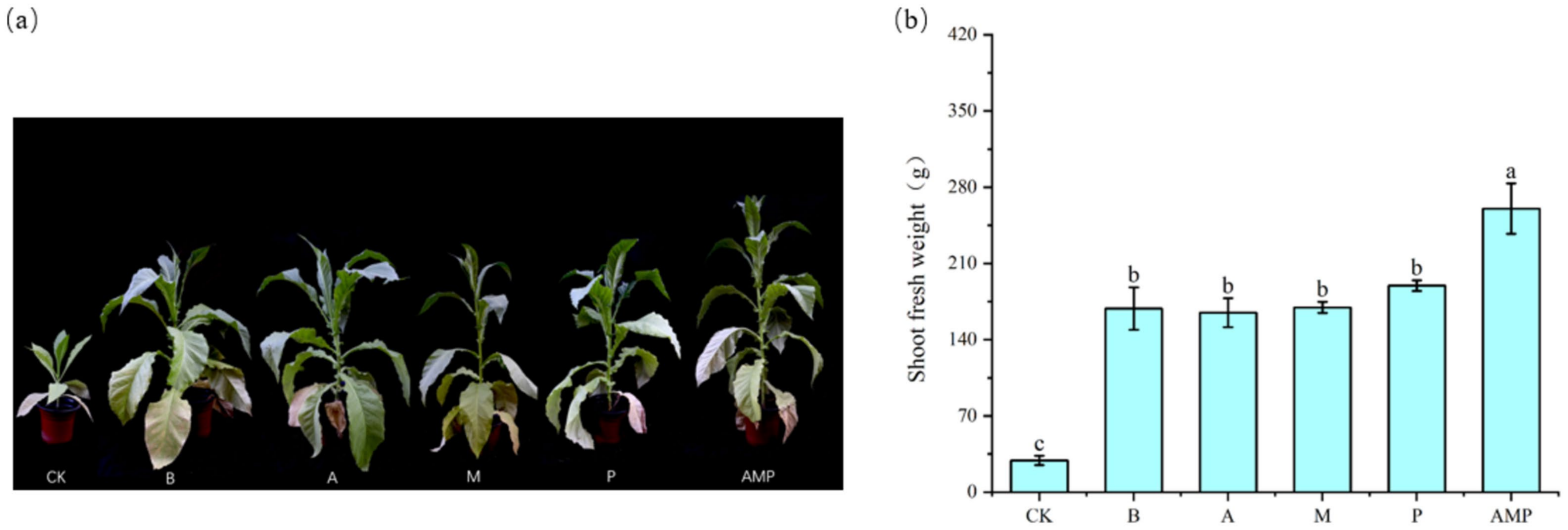
Phenotypic map of rhizosphere soil bacteria and fresh weight of aboveground parts after 6 weeks. CK was not inoculated with inter-root soil bacteria; B: *Bacillus cereus*, A: *Arthrobacter* sp., M: *Massilia* sp., P: *Pseudomonas* sp., APM is a consortium of three bacterial strains. **(A)** Phenogram of rhizobial soil bacteria after six weeks of planting. **(B)** Fresh weight of shoot after six weeks of planting.

When pathogens activate the ISR pathway, plant resistance to disease increases, but plant growth, development, and seed production decline (Vos et al., 2015; Van Hulten et al., 2006). However, rhizosphere soil bacteria B can also promote the growth and development of plants after inducing plants to activate the ISR pathway (Pieterse et al., 2014; Yan et al., 2022). Therefore, we returned the three isolated rhizosphere soil bacteria to the tobacco rhizosphere soil to evaluate their impact on plant growth. We found that all bacterial strains, including strain B, had no negative effect on the fresh weight of aboveground parts of tobacco plants and even promoted an increase in the net photosynthetic rate of plants (Figures 6A,B, 7A,B). The net photosynthetic rate and aboveground fresh weight of tobacco plants increased most significantly under the treatment of a combination of three rhizosphere soil bacteria.

### 3.5 Formation of soil borne legacy

**Figure 8:**
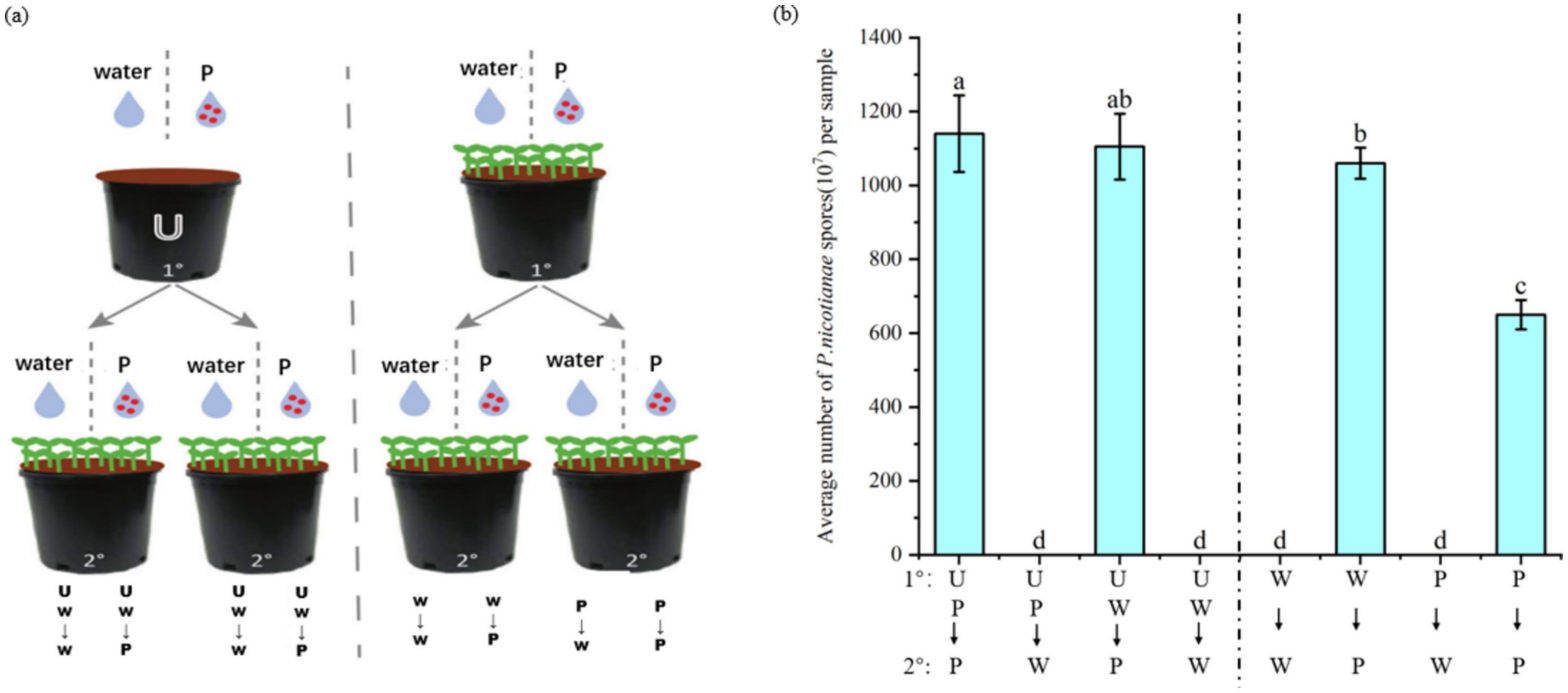
Effect of soil-mediated tobacco black shank infection on disease resistance of subsequent plant populations. The first batch (1°) of tobacco seedlings or un planted soil (U) was treated with tobacco black shank pathogen suspension (P) or sterile water (W). The second batch of plants (2°) were inoculated with pathogens or treated with sterile water when four leaves were old. After 2 weeks of treatment, tobacco was harvested to measure the number of pathogen spores at the lesion and to quantify the severity of the disease. **(A)** Schematic diagram of the experiment on the effect of soil-mediated infection with tobacco black shank disease on subsequent plant resistance. **(B)** Average number of *P. nicotianae* spores after experimental treatments.

ISR can enhance plant defense against future pathogens or pest attacks (Pieterse et al., 2014), but it is unlikely to completely cure plants infected by pathogens. To verify whether plants will recruit a batch of specific beneficial bacteria in the rhizosphere soil to help subsequent plants (such as plant offspring) grow in the same soil to resist diseases after pathogen attacks. In this experiment, the soil was pretreated by planting tobacco plants infected with *P. nicotianae* and treated with sterile water. The second batch of tobacco was planted on different pretreated soils, and the tobacco was infected with *P. nicotianae*. The degree of disease infection was quantified by measuring the number of spores. The experimental flow chart is shown in Figure 8A. A control soil without plants was also set up during the preparation phase of the experiment. The soil treatment method followed the above, including sterile water and *P. nicotianae* spores.

The resistance of tobacco plants grown in the soil without tobacco plants infected by black shank disease was similar to that of tobacco treated with sterile water and pathogen spores. The specific performance was that there was no notable difference in the number of spores in the UP–P and UP-W groups. Tobacco seedlings grown in soil pretreated by plants infected with *P. nicotianae* were more resistant to tobacco black shank infection than plants grown in soil treated with sterile water. Specifically, the number of pathogen spores in the P–P group was significantly lower than in the W-P group (Figure 8B). These results indicate that tobacco plants will enrich a batch of specific bacteria in the soil to form soil heritage after being infected by *P. nicotianae* to help subsequent plant populations growing in the same soil resist pathogen infection.

## 4 Discussion

### 4.1 Changes of bacteria in tobacco rhizosphere soil under pathogen infection

In this experiment, the 10 high-ranking bacterial phyla and genera of abundance in tobacco rhizosphere soil were selected for analysis by monitoring the changes in rhizosphere soil bacteria in different periods of tobacco. Among them, the dominant bacteria phyla of each treatment were *Proteobacteria*, *Actinobacteria*, and *Acidobacteria*. Still, the proportion of each phylum varied under different treatments. Among them, *Actinobacteria*, *Proteobacteria*, and *Acidobacteria* were the dominant bacteria in different soil types (Zhao et al., 2014; Li and Wang, 2013; Han et al., 2014). *Proteobacteria* and *Acidobacteria* may be used as indicators of soil nutrient status (Sun et al., 2013). *Acidobacteria* are ubiquitous in soil (Neufeld and Mohn, 2005), mostly survive in nutrient-poor soil environments (Dion, 2008), and the abundance of *Acidobacteria* is negatively correlated with the availability of carbon (Fierer et al., 2007). *Proteobacteria* are considered the most prevalent phylum in the world (Neufeld and Mohn, 2005). *Actinobacteria* is a phylum with a large content in the soil. *Actinobacteria* are the main bacteria used for antibiotic production to adjust the number of *Actinobacteria* in soil. The proportion of antagonistic *Actinobacteria* plays an important role in adjusting the ecological balance of soil microorganisms (Zhang et al., 2010).

The abundance of *Proteobacteria* in the rhizosphere soil of tobacco plants infected by *P. nicotianae* increased significantly with the increase over time compared with healthy tobacco rhizosphere soil bacteria. This result indicated that the pathogen would cause changes in tobacco rhizosphere soil bacteria after infecting tobacco. Specifically, tobacco recruits *Proteobacteria* in the rhizosphere to help host plants resist diseases.

### 4.2 Effects of rhizosphere soil bacteria on host plants

This experiment showed that after tobacco is infected by *P. nicotianae*, a group of soil bacteria will be specifically recruited in the rhizosphere soil to help the host plant resist disease. Here, we selected two rhizosphere soil bacteria with high abundance and one with low abundance: *Pseudomonas* sp., *Massilia* sp., and *Arthrobacter* sp., which can form a consortium to help tobacco resist disease. Studies have found that after a pathogen infects tobacco, it sends signals to plant roots to promote the growth of specific bacteria in the rhizosphere soil. The promoted rhizosphere soil bacteria play a systematic role in forming biofilms. This indicates that these bacteria also play a synergistic role in the rhizosphere soil bacteria community as a consortium to help promote plant growth and help host plants resist diseases.

After *P. nicotianae* infects tobacco plants, they can promote the growth of specific members of their microbiomes to increase disease resistance. Recent studies on *Arabidopsis* and wheat have supported this view, leading to changes in the rhizosphere soil bacterial community of plants after pathogen infection (Berendsen et al., 2018; Dudenhffer et al., 2016). Studies have shown that *Pseudomonas* sp. NXHG29 can reduce the occurrence of tobacco black shank and bacterial wilt along with *Pseudomonas* sp. ISR requires the number of bacteria to maintain a threshold population level of 10^5^ CFU/g (Raaijmakers et al., 1996; Ma et al., 2018). The reduction of rhizosphere microbial community diversity and the enrichment of some bacterial communities make plants more susceptible to pathogens (Hamid et al., 2017; Van Elsas et al., 2012). However, the influence of the relative abundance of components in synthetic communities cannot be excluded (Lebeis et al., 2015; Erbs et al., 2010). The presence of low-abundance bacteria also helps accommodate other bacterial types recruited by root exudates. This process may be beneficial for plant growth, stress tolerance, and the overall healthy growth of plants (Saravanakumar et al., 2018).

### 4.3 Formation of a soil -borne legacy

ISR can enhance the defense ability of plants against pathogens or insect attacks (Pieterse et al., 2014). Still, ISR is unlikely to completely cure plants that pathogens have infected. In nature, plants accumulate pathogens in their surrounding soil, eventually harming them. This phenomenon is called the negative feedback of soil (Van der Putten et al., 1993; Bever et al., 2012). The accumulation of this soil-borne disease is devastating in agriculture, but it can be alleviated using crop rotation. The ability to inhibit disease in soil usually occurs after an outbreak of disease (Weller et al., 2002; Berendsen et al., 2012). This process indicates that when pathogens attack plants, they recruit a group of bacterial communities to protect them from disease erosion. The protective ability of plants to utilize their root bacteria depends on the genotype of plants (Wintermans et al., 2016; Pieterse et al., 2016). Therefore, we hypothesized that when pathogens infect plants, the relevant protective bacteria recruited by plants in the rhizosphere soil can help subsequent plants growing in the same soil resist diseases. This experiment showed that after plants are infected by pathogens, they indeed recruit a batch of specific rhizosphere soil bacteria, forming a soil legacy that eventually helps subsequent plant populations growing in the same soil resist diseases. Although the direct impact of chemical substances produced by plants cannot be ruled out, this legacy effect is likely to come from bacteria. In other words, after a pathogen infects a plant, it causes the plant to recruit a group of specific bacteria in the rhizosphere soil to help the plant resist disease, and these bacteria will also protect subsequent plant populations growing in the same soil from infection by pathogens.

To sum up, the composition of the plant rhizosphere soil bacteria community changes after plants are infected by oomycete pathogens. Host plants recruit particular beneficial bacteria that induce disease resistance and promote plant growth, thereby maximizing the protection of offspring growing in the same soil from disease.

## Data Availability

The datasets presented in this study can be found in online repositories. The names of the repository/repositories and accession number(s) can be found in the article/Supplementary material.
