# Supplementary material for: Black shank-mediated alteration of the community assembly of rhizosphere soil bacteria in tobacco
Source: Front Microbiol. 2024 Oct 23;15:1428284. doi: 10.3389/fmicb.2024.1428284 (PMC11538049; doi:10.3389/fmicb.2024.1428284)
Supplement: Supplementary file 1 [file Table_1.DOCX]

# Supplementary Information

## Attachment


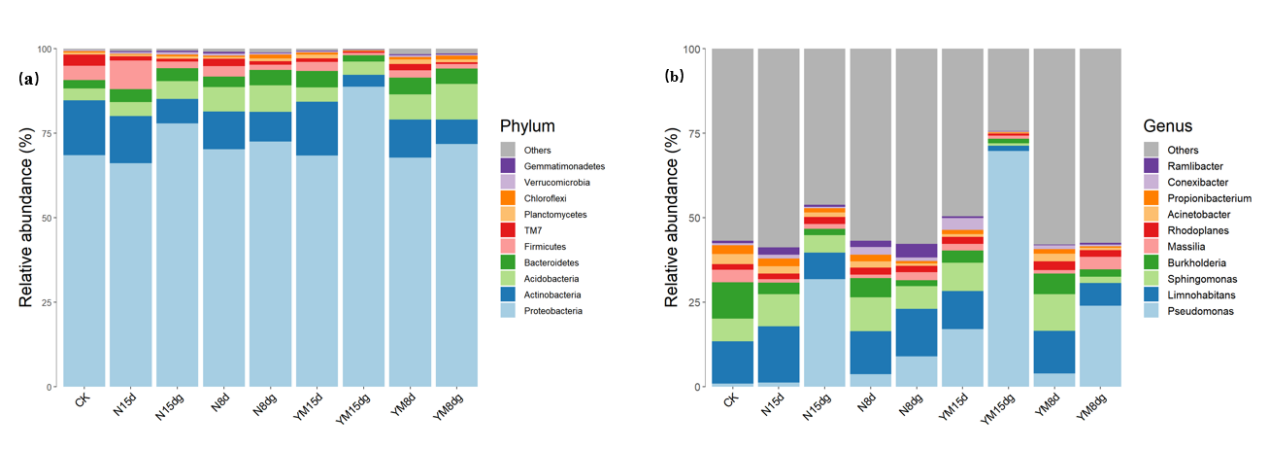


Attachment 1 Species composition of microbial communities at the level of phylum and genus

Note: Note: CK refers to non-planted soil; N15d is the non-rhizosphere soil of tobacco on the 15th day of health; N15dg is the tobacco rhizosphere soil on the 15th day of health; N8d is the non-rhizosphere soil of tobacco on the 8th day of health; N8dg is the tobacco rhizosphere soil on the 8th day of health; YM15d is the non-rhizosphere soil of tobacco on the 15th day after pathogen infection; YM15dg is the tobacco rhizosphere soil on the 15th day after being infected with the pathogen; YM8d is the non-rhizosphere soil of tobacco on the 8th day after being infected with the pathogen; YM8dg is the tobacco rhizosphere soil on the 8th day after being infected with the pathogen.

**
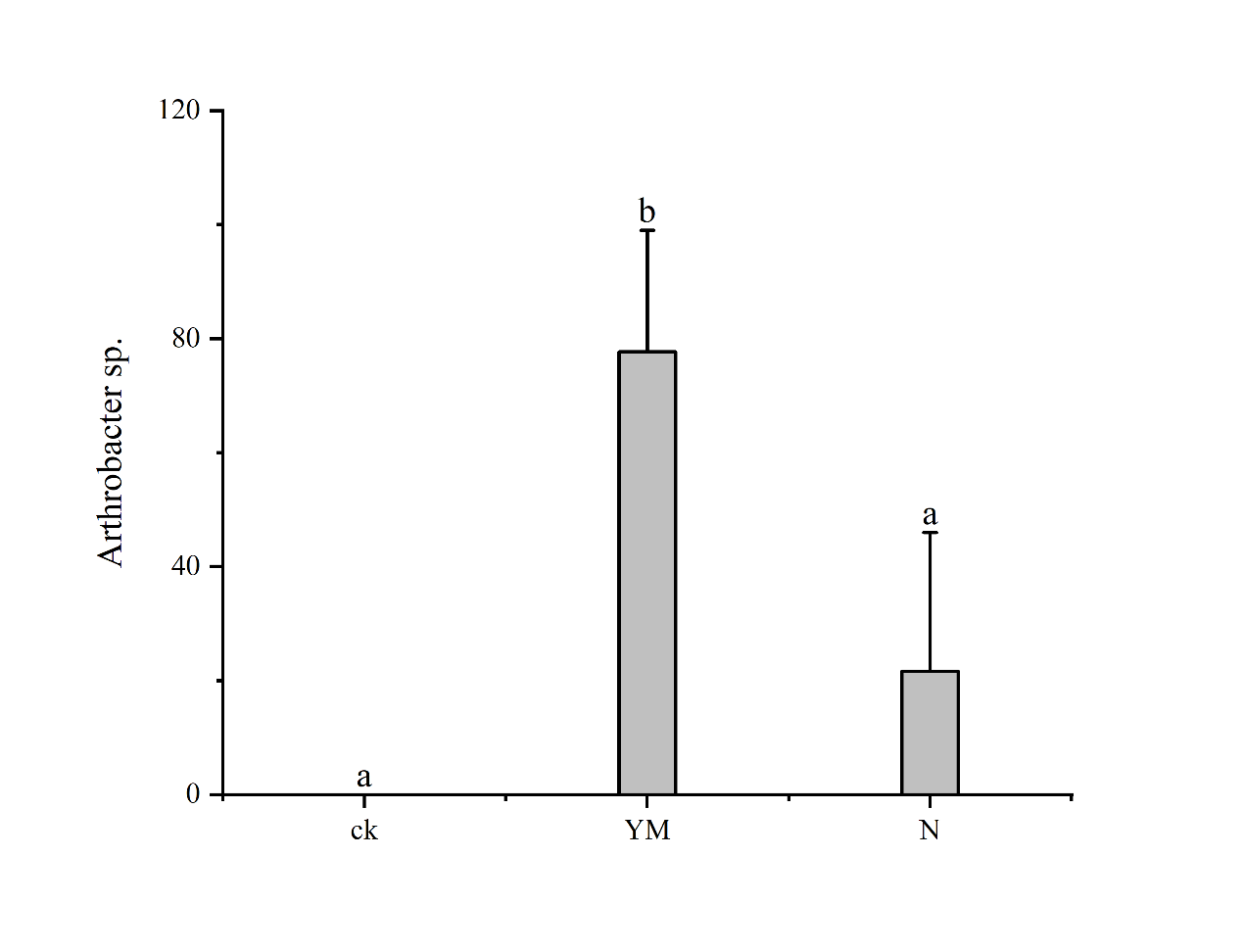
**

Attachment 2. Abundance of *Arthrobacter sp.* under pathogenic infestation versus normal growth in tobacco roots

Note: ck is ungrown tobacco soil, YM is tobacco inter-root soil infected with pathogen, and N is normal growing tobacco inter-root soil.

**Table S1 Taxonomy of isolates collected from rhizospheres of P. nicotianae-infected tobacco plants.**

| **Phylum** | **Genus** | **Number of isolates** |
| --- | --- | --- |
| Actinomycetota | *Arthrobacter* | 7 |
| Actinomycetota | *Aestuariimicrobium* | 1 |
| Actinomycetota | *Kocuria* | 6 |
| Actinomycetota | *Micrococcus* | 7 |
| Actinomycetota | *Microbacterium* | 19 |
| Actinomycetota | *Nocardioides* | 2 |
| Actinomycetota | *Pseudarthrobacter* | 2 |
| Actinomycetota | *Streptomyces* | 9 |
| Bacteroidetes | *Chryseobacterium* | 3 |
| Bacteroidetes | *Dyadobacter* | 2 |
| Bacillota | *Aerococcus* | 1 |
| Bacillota | *Bacillus* | 106 |
| Bacillota | *Fictibacillus* | 3 |
| Bacillota | *Geobacillus* | 1 |
| Bacillota | *Paenibacillus* | 1 |
| Proteobacteria | *Alcaligenes* | 1 |
| Proteobacteria | *Acidovorax* | 2 |
| Proteobacteria | *Comamonas* | 1 |
| Proteobacteria | *Delftia* | 2 |
| Proteobacteria | *Lysobacter* | 1 |
| Proteobacteria | *Massilia* | 6 |
| Proteobacteria | *Pseudomonas* | 4 |
| Proteobacteria | *Pseudoxanthomonas* | 9 |
| Proteobacteria | *Ralstonia* | 17 |
| Proteobacteria | *Ramlibacter* | 2 |
| Proteobacteria | *Rhizobium* | 2 |
| Proteobacteria | *Shinella* | 3 |
| Proteobacteria | *Stenotrophomonas* | 2 |
| Proteobacteria | *vibrio* | 2 |
|  | **Total** | **224** |
